# Health, Stress and Technologies: Integrating Technology Acceptance and Health Belief Models for Smartphone-Based Stress Intervention

**DOI:** 10.3390/healthcare11233030

**Published:** 2023-11-23

**Authors:** Giulia Paganin, Simona Margheritti, Naima Z. Farhane-Medina, Silvia Simbula, Greta Mazzetti

**Affiliations:** 1 Department of Educational Science, University of Bologna, 40126 Bologna, Italy; greta.mazzetti@unibo.it; 2 Department of Psychology, University of Milano-Bicocca, 20126 Milano, Italy; simona.margheritti@unimib.it (S.M.); silvia.simbula@unimib.it (S.S.); 3 Bicocca Center for Applied Psychology, Department of Psychology, University of Milano-Bicocca, 20126 Milano, Italy; 4 Department of Psychology, University of Cordoba, 14071 Cordoba, Spain; nfarhane@uco.es; 5 Maimonides Biomedical Research Institute of Cordoba (IMIBIC), 14004 Cordoba, Spain; 6 Reina Sofia University Hospital, 14004 Cordoba, Spain

**Keywords:** technology acceptance model, health belief model, technology usage, smartphone-based intervention, stress management, well-being promotion

## Abstract

Work-related stress significantly jeopardizes employees’ physical and mental health due to the considerable time they spend at work. Smartphone-based interventions provide a promising solution, eliminating traditional face-to-face interventions’ barriers. However, the elements that influence workers’ intentions to use this still remain unexplored. This study explores the link between health belief model (HBM) and technology acceptance model (TAM) factors. In this study, 336 Italian workers (64% female) answered an online questionnaire. We employed a structural equation model (SEM) to analyze the data. The results unveiled an indirect relationship: individuals perceiving health risks were more inclined to use stress-management apps, mediated by perceived utility (PU). This study underscores the significant potential of integrating the HBM with the TAM in predicting users’ preparedness for smartphone-based health interventions. These findings not only hold substantial value but also illuminate a path forward for professionals and organizations, offering insights to tailor and optimize smartphone tools for stress management and the promotion of workplace well-being. Ultimately, this research paves the way for the cultivation of healthier work environments, marking a noteworthy contribution to the field.

## 1. Introduction

Workplace stress is recognized as a significant risk factor for various mental and physical health issues among employees [1]. Experiencing stress can have a negative impact on employee satisfaction and performance [2,3]. As a result of the substantial influence on employee well-being, productivity, and organizational success, the analysis of workers’ stress has gained major relevance [4]. In recent years, companies have increasingly recognized the importance of promoting employee health [5]. Such an analysis is critical for the creation of solutions that effectively reduce stress while also increasing a company’s productivity and efficiency [6]. From a preventive perspective, interventions should be implemented before stress-related health problems develop that can contribute to chronic disease. However, research shows that traditional personal interventions often have limited effectiveness [7]. To address this challenge and improve participation in well-being and stress-management interventions, the implementation of digital mental health treatments in the workplace has been proposed. Consequently, a paradigm shift from traditional face-to-face interventions to digital modalities has been observed over the last decade [8]. The importance of user acceptance has been highlighted in the scientific literature as a crucial factor for the development of any technology [9]. Therefore, it is critical to understand workers’ intentions to use and accept technology before introducing innovations in organizations, such as a mobile app to manage stress and promote well-being.

As a result, numerous theoretical models have been developed to analyze and explain technology acceptance and related behaviors. Particularly, the technology acceptance model (TAM) is a widely accepted model to predict and explain the use of technologies, which has shown positive results in several studies [10]. However, this model was not specifically formulated to investigate the use of health-related technologies [11]. For this reason, we also chose to consider the health belief model (HBM) proposed by Rosenstock et al. [12] that provides a solid framework for understanding people’s health behaviors and attitudes.

Therefore, the aim of the present study is to integrate these two models in order to better explain the intention to use a smartphone app to manage stress. Indeed, the integration of these models allows for two complementary aspects. If the TAM is indeed very good at predicting an individual’s use of an information system, the HBM seeks to explain the factors that may influence the user’s engagement with a health behavior, such as a stress-management app.

To date, to the best of our knowledge, there are no studies that integrate these models with respect to the use of smartphone-based stress interventions in work settings.

## 2. Theoretical Background and Hypothesis Development

### 2.1. Smartphone-Based Interventions for Stress Management

Increased and chronic stress adversely impact worker satisfaction and performance [2,3], leading to various disorders and increased absenteeism, turnover, and reduced productivity [1,2,3,13]. Consequently, organizations seek effective approaches to manage work-related stress and promote well-being among employees [14]. However, resource limitations hinder some companies from developing adequate stress interventions, prompting research into alternative methods [8]. Shifting from traditional face-to-face approaches, technology-mediated interventions, particularly via smartphones, offer continuous monitoring, accessible support, anonymity, and personalized interventions catering to individual needs. For example, employees may begin and complete the self-guided intervention at their own speed, selecting the information that best pertains to their present concerns via a digital pathway. Self-guided applications with short and basic activities may thus be introduced into employees’ everyday routines, increasing the chances of the desired behavior occurring. Both the person and the organization profit from this [8,15,16]. Mobile apps for mental health monitoring have the potential to treat burnout, stress, depression, and anxiety [17,18,19,20]. However, despite some encouraging results, there is limited evidence on the effectiveness of smartphone-based interventions [16].

Research highlights some barriers to the successful implementation and effectiveness of mobile-based treatments in the workplace, such as a high dropout rate due to limited employer participation in programs [21,22]. However, the number of studies regarding the acceptance of smartphone-based interventions in the workplace is still growing. Indeed, investigating the employee adoption of new technologies, particularly smartphone applications, is one way to prevent employee nonparticipation in wellness and health initiatives. Therefore, it seems crucial to recognize that technology acceptance is a prerequisite for effective technology adoption in an organization.

### 2.2. Technology Acceptance

The literature on technology adoption explores how people’s opinions impact both their intentions to utilize technology and its actual use, which is especially important given the proliferation of new technologies [23]. The importance of user approval is widely addressed in the scholarly literature [9]. Although smartphone-based interventions have been shown to improve well-being and stress management, the findings are still mixed [24]. Before implementing innovations in the workplace, such as a stress-management mobile app, a comprehensive understanding of workers’ intentions and acceptance is required. Various theoretical models have been presented during the last three decades to study and explicate technology adoption and associated behavior. The TAM, developed by Davis [11], is one prominent model that extends the established theoretical framework of Fishbein’s [25] and Fishbein and Ajzen’s [26] theory of reasoned action. The TAM’s main variables are perceived utility (PU), which reflects the belief in the system’s ability to improve work performance, and the perceived ease of use (PEOU), which measures the perceived ease of using a certain tool. Intention to use (INT) is the desire to employ a specific technology [27]. According to the TAM, PU and PEOU directly influence adoption, which in turn influences INT. As a result, the inclination to use a stress-management intervention is closely related to how employees view its usefulness and ease of use. We chose the TAM over Davis’s [11] unified theory of acceptance and use of technology (UTAUT; [28]) because of its simplicity and efficacy in explaining technology adoption through the crucial components of PU and PEOU. The UTAUT, on the other hand, necessitates assessing a specific technology within a defined population, making it less appropriate for examining early assessments related to personality traits in the larger context of general technology adoption. This model might be improved further by including external factors to achieve a more thorough knowledge of technological adoption and application [29,30]. Recognizing that elements other than PU and PEOU may impact acceptance and the desire to adopt a particular technology [31] is a recurring topic in these advancements. As a result, we chose to look at the impact of workers’ health beliefs regarding stress-related health conditions on perceived utility and the intention to use an app for stress management and well-being promotion Moreover, the TAM has received extensive scientific support for its core variables. Additionally, it is important to highlight that the original formulation of the TAM also incorporated the dimension of attitude toward technologies, serving as a mediator between perception and behavioral intention. However, subsequent empirical findings by Davis, Bagozzi, and Warshaw [27] revealed a nonsignificant effect in the link between attitude and behavioral intention. They clarified this outcome by noting that individuals might use a technology even without a positive attitude towards it if they perceive it as enhancing productivity. Consequently, the final TAM model, as proposed by Davis, Bagozzi, and Warshaw [27], excluded the attitude construct.

Starting from these suggestions, we hypothesized the following:

**H1a:**  *PU is associated with the INT a smartphone app for stress management.*

**H1b:**  *PEOU is associated with the INT a smartphone app for stress management.*

**H1c:**  *PEOU is associated with PU.*

In addition, the TAM postulates that PU is influenced by ease of use, suggesting that technologies that are perceived as more user-friendly are considered more relevant [23]. Therefore, we believe that the INT an app for managing stress is indirectly influenced by perceptions of ease of use, in part because perceptions of usefulness increase.

**H1d:**  *PEOU is associated with the INT a smartphone app for stress management, also through its PU.*

Subsequent developments of this model have revealed that other external variables might aid in understanding technology adoption and, therefore, application [24,25]. A common feature of these adjustments is the recognition that characteristics other than PU and PEOU might impact acceptance and intention to use a specific technology [26], even though PU appears to be one of the most important predictors of INT [11]. For this reason, we decided to investigate the association of beliefs related to one’s health versus the susceptibility and perceived severity of stress-related symptoms on perceived usefulness and intention to use an app for stress management and wellness promotion.

### 2.3. Integrating TAM and HBM

The technology acceptance model (TAM) analyzes, from a technological standpoint, the intention to use smartphone-based interventions based on perceived their utility and simplicity of use. The health belief model (HBM), on the other hand, investigates an individual’s subjective appraisal of vulnerability and susceptibility to stress-related health risk. Originally intended to predict treatment responses in acute or chronic diseases [27], the HBM has now been broadened to anticipate general health behavior [28,29]. The HBM is widely used and effectively elucidates numerous health behaviors across distinct demographics [30].

Fundamentally, the HBM claims that people do not engage in health-related activities unless they are psychologically prepared, which occurs most often when they perceive vulnerability to illness. Belief in health risks predicts participation in health-related activities [27]. Disease susceptibility and disease severity are two categories of perceived health risks. Disease susceptibility reflects beliefs about the likelihood of illness, whereas disease severity assesses the perceived seriousness of the illness, taking into account medical, clinical, and social consequences [31]. A perceived high level of health risk might induce behavioral modifications such as the use of a stress-management smartphone app. The TAM and the HBM were used in previous research to investigate health-related Internet use. Recognizing their inadequacy in isolation, later studies tried to combine them [30,32,33,34,35]. Mathieson [36] noticed the applicability of both theories in forecasting user behavior, emphasizing the TAM’s simplicity and the HBM’s comprehensiveness [37]. Yun [38] developed an integrated model that incorporated the TAM [11], the HBM [27], the theory of reasoned action [39], and the TPB [40]. Kim and Park [41] developed the “Health Information Technology Acceptance Model” (HITAM) to fit digital health interventions after seeing commonalities in key principles across these theories. Melzner et al. [42] expanded on these existing features to offer a theoretical model for measuring mobile health program adoption in workplaces. The key aspects of the two models, in our opinion, can be connected and reliably predict the desire to use a smartphone app for stress management. First, we hypothesize that people who are more vulnerable to stress or perceive a greater impact of stress will be more motivated to use a smartphone app. Indeed, a person who believes that he or she is susceptible to stress may be more motivated to look for solutions to alleviate the negative effects of stress, such as the use of an app.

Moreover, they will have a stronger sense of the benefits of stress prevention apps. In other words, cognitive beliefs related to a specific technology become central to an individual’s perceived health risk or health awareness.

Finally, this perception may lead to an increased likelihood of using such applications to prevent and thereby alleviate the negative effects of stress.

This is in line with Ahadzadeh et al. [30], who empirically demonstrated the direct relationship between perceived health risk and the use of the Internet for health management through PU. Also, Melzner et al. [42] assert that, while perceived vulnerability and perceived gravity offer an incentive for taking preventative measures, perceived usefulness may impact the course of action, influencing the intention to use. As a result, these individuals should make greater use of the technology in question. Thus, we hypothesized the following:

**H2a:**  *PHRs are associated with the INT a smartphone app for stress management.*

**H2b:**  *A PHRs is associated with the PU of a smartphone app.*

**H2c:**  *PHRs are associated with the INT a smartphone app for stress management through PU.*

**Figure 1:**
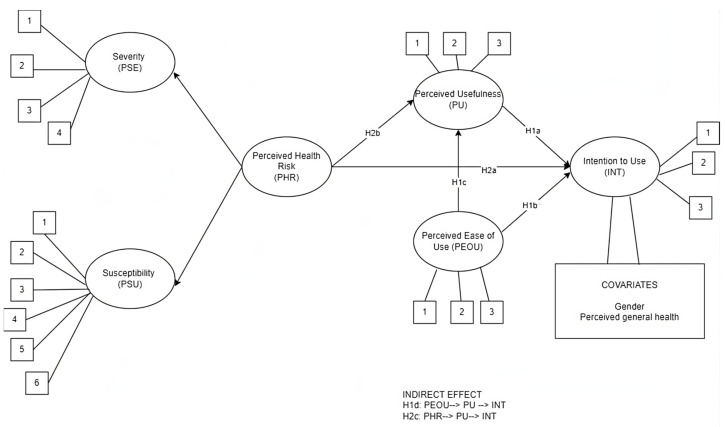
The hypothesized model.

The final hypothesized model is reported in Figure 1.

## 3. Materials and Methods

### 3.1. Context and Participants

Data collecting took place at three Italian businesses in northern Italy in July 2020. The Human Resources Department issued an email to all workers informing them of the opportunity to participate in this survey. This e-mail said that participation in the research was purely voluntary, that the study was wholly managed by the University of Milan-Bicocca, and that the company would not have access to the workers’ responses. After eight days, a second e-mail was sent to employees, reminding them of their opportunity to participate in the survey.

The participants were required to read the informed consent forms before completing the questionnaire. The data collection was conducted in conformity with the ethical standards established by the Declaration of Helsinki and was authorized by the Ethical Committee of the University of Milano-Bicocca (Prot. N. RM-2020-312).

In total, 336 responses were included in our dataset. Among them, 64.9% were female and 35.1% were male; the mean age was 37.28 years (SD = 10.78). A total of 20.8% had a high school diploma; 56% had a bachelor’s or master’s degree; 19.3% had a higher educational level; and only 3.9% had a different or lower study level. The mean level of seniority at their job was 12.8 years (SD = 10.10). Regarding health status (PGH), 6.5% reported suffering from chronic psychophysical health conditions (e.g., panic attacks, psychosomatic symptoms, chronic headaches, etc.). Concerning previous experience with smartphones, almost all participants (98.5%) were familiar with using a smartphone, and 13.7% had used a stress-management or well-being-promotion app in the past.

### 3.2. Measures

The TAM dimension of PEOU was assessed through three items (e.g., “It will be easy to use the app”), PU was measured with four items (e.g., “The presented app could help me improve my work-related well-being”), and INT was measured through two items (e.g., “I would like to try the presented app”). The items were assessed through a Likert scale from 1 = strongly disagree to 5 = strongly agree. The items were all taken from previous studies [43,44,45].

The PHR contains two subdimensions: perceived susceptibility to chronic diseases and perceived severity of chronic diseases. Perceived susceptibility (PSU) to chronic diseases was measured using 6 items adopted from Kim and Park [41] and Bryan et al. [46]. Perceived severity (PSE) to chronic diseases was measured using 4 items adopted from the Kim and Park study [41]. All items of these constructs were rated on a 5-point Likert-type scale from 1 = strongly disagree to 5 = strongly agree.

### 3.3. Data Analysis

Descriptive statistics, Pearson correlations, and Cronbach’s alpha coefficients were calculated using SPSS v.28 (IBM, Armonk, NY, USA). The objective was to observe the sample characteristics, the correlations between variables, and the scale reliability. Moreover, the data were checked for outliers and normality distribution. The skewness levels (ranging from 0.32 to −0.38) and kurtosis values (ranging from 0.69 to −0.68) were acceptable [47].

To test our hypotheses, we performed a full structural equation model (SEM) using Mplus version 8 (Muthén & Muthén, Los Angeles, CA, USA). The SEM offers three prominent benefits when contrasted with conventional multivariate methodologies: (1) a clear and meticulous evaluation of measurement errors, (2) the ability to estimate latent (unobservable) variables through observed variables, and (3) the capacity for model testing, wherein a structural framework can be defined and subsequently evaluated for its compatibility with the collected data. The model included the direct effect of PHR, PU, and PEOU on the INT to use a smartphone app; the indirect effect of PEOU on INT, via PU; and the indirect effect of PHR on the INT a smartphone app via its PU. Moreover, we added the effect of gender and perceived general health as covariates.

To assess the model’s goodness-of-fit, we used the statistic criteria listed below: the non-significant χ^2^ value (this statistic suggests that if χ^2^ is non-significant, the model fits the data); the root mean squared error of approximation (RMSEA; values smaller than 0.08 indicated an acceptable fit); the comparative fit index (CFI) and the Tucker–Lewis index (TLI; values between 0.90 and 0.95 showed an acceptable fit); and the standardized root mean square residual (SRMR; values smaller than 0.08 indicated a proper fit). Through bootstrapping procedures, it is possible to perform repeated subsample simulations from an original dataset. For this reason, the bootstrapping method was conducted to assess the significance of the hypothesized indirect effects.

## 4. Results

### 4.1. Descriptive Statistics

The means, standard deviations, and correlations between the study variables are reported in Table 1. All significant relationships among variables were in the expected direction. Furthermore, as indicated along the table diagonal, all scales reported an internal consistency value (Cronbach’s alpha) exceeding the criterion of 0.70 [48].

### 4.2. Confirmatory Factor Analysis (CFA)

We used the maximum likelihood method in a CFA to assess the structural validity of our measures. The results of the CFA (see Table 2) show that the factor model including perceived chronic disease susceptibility and perceived chronic disease severity loaded on the PHR, PEOU, PU, and INT of smartphone apps fit our data best compared with other solutions (Table 3). This finding supports the discriminant validity of the measures employed in the current study.

### 4.3. Direct Effects

The hypothesized model reported an adequate fit to our data, with χ^2^ (58) 140–717, *p* = 0.000, CFI = 0.98, TLI = 0.97, RMSEA = 0.06, 90% CI 0.06, 0.07, SRMR = 0.05.

The results indicated a strong and positive relationship between the PU and INT for smartphone apps (H1a). Additionally, the PEOU was not found to have a significant association with the INT (H1b). However, our results confirm the association with the PU (H1c).

**Figure 2:**
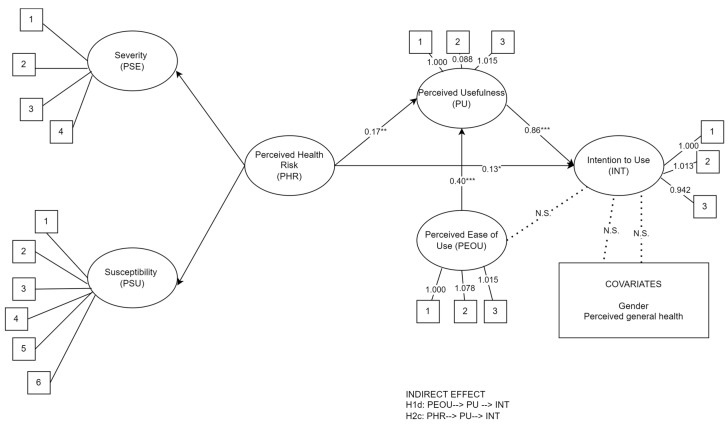
Direct effects. Note. * *p* < 0.05; ** *p* < 0.01; *** *p* < 0.001. Straight arrows indicate significant effects; dotted arrows indicate non-significant (N.S.) direct effects. Numbers in square indicate the observed variables. Ovals indicate latent variables; squares indicate observed variables.

According to the effect of perceived risk, our results described a significant association between the PHR and the INT for smartphone apps (H2a) and PU (H2b). Finally, regarding covariates, neither gender nor perceived general health had an influence on the intention to use smartphone apps (INT). The direct effects are reported in Figure 2.

### 4.4. Mediation Analysis

Concerning the mediations, Table 3 presents the indirect effects. The findings demonstrated that PU played a mediating role in the association between PEOU and INT (β = 0.30, *p* < 0.000) (H1d). Interestingly, there was a significant relationship between PHR and INT when PU acted as mediator (β = 0.14, *p* < 0.005) (H2c).

## 5. Discussion

In recent decades, there has been a significant and continuous increase in interest in the prevention of work-related stress. The recent literature has emphasized the potential role of new technologies in addressing this problem, which has led to a shift in the framework for implementing such interventions, focusing on adaptations to technological strategies [49,50,51,52]. Apps for mobile devices have begun to be utilized for workplace health promotion. However, the elements that influence the intention to use an app in the workplace remain largely unexplored. Among the various theories commonly used to address this issue, the TAM and the HBM stand out for their adequacy. Therefore, the aim of this study was to integrate the TAM and the HBM in the context of smartphone-based interventions to manage work-related stress. While the combination of the TAM and the HBM has been previously used to explain health-related Internet use [30,53,54], there is limited research applying both models to mobile applications for managing stress and promoting well-being in the work context. Therefore, the potential contribution of this study is to identify the relevant variables that may influence the use of a mobile app and reduce the likelihood of developing chronic stress and other associated health risk factors. In this way, this study aims to improve the effectiveness of smartphone-based interventions and contribute to the overall well-being of individuals suffering from work-related stress, both in terms of alleviation and prevention.

In general, the results found were consistent with the hypotheses. The hypothesis on the TAM was partially confirmed. On the one hand, a significant association was found between PU and the intention to use a smartphone app for stress management (H1a), and PEOU was associated with PU (H1c). These results are consistent with the previous literature. Indeed, several studies identified the potential influence of PU as one of the factors positively affecting subjectively measured mobile health app use [55,56,57,58], consistent with Davis’s [11] theory for the TAM. However, our results did not confirm the direct effect of PEOU on INT (H1b [11,22,59,60,61,62]). Again, our results are partially in line with previous studies. For example, studies by Jung & Loriaphr [59] and Wahyuni et al. [63] did not confirm the positive and significant effect of PEOU on INT, suggesting that an app’s ease of use is not always an issue in the utilization of digital interventions. This finding suggests that perceived usefulness plays a more critical variable on intention to use smartphone-based interventions. Moreover, the results taken together could also indicate that the association between PEOU and INT may be mediated by PU, as hypothesized, and confirmed in this study (H1d). However, the conflicting results of other studies suggest that such associations may vary depending on the specific context and user population studied. Probably, the workplace, with its specific characteristics, elicits different acceptability criteria than private health apps [60]. Therefore, further study of the target population is needed to obtain specific results and to gain a deeper understanding of the relationships between these variables.

Regarding the integration of the TAM and the HBM, all three hypotheses were confirmed. There was a significant relationship between the PHR and INT of the stress-management app (H2a) and PU (H2b), and a mediating role of PU in the relationship between PHR and intention to use (H2c). These findings are consistent with previous studies that found a positive relationship between PHR, PU, and INT for health-related digital tools [32,41,54], suggesting, in agreement with the HBM [12,61], that health risk belief predicts the likelihood of engaging in healthy behaviors. In the research on health behavior and the adoption of smartphone-based apps for stress management and well-being promotion in the workplace context, the relationship between perceived severity and susceptibility as general motivators and perceived utility as dictating the chosen course of action is quite innovative. Indeed, although the TAM and the HBM have previously been integrated to explain the use of digital tools for health promotion, this study expands previous insights to assess the intention to use apps for well-being promotion and stress management in the work context. They also emphasize the importance of PU as an important factor to consider when developing and promoting smartphone apps to improve health behaviors, such as stress management in the workplace.

In reality, when users perceive a health risk, they can opt to try a smartphone-based solution to improve their health management. In other words, perceiving a greater risk to one’s health leads one to perceive the smartphone-based intervention as more useful and this leads to an increased intention to use it. As mentioned earlier, work-related stress brings a number of negative consequences to workers’ health that can lead to chronic complications and affect various aspects of a person’s life [5,50,62,64]. Companies and organizations are looking for ways to mitigate these consequences for their employees for a variety of reasons (improving productivity, promoting job satisfaction, etc.) [5]. Given the lack of economic and human resources to implement preventive measures in the workplace, new technologies have emerged as an interesting alternative [7]. However, studies that have examined the effectiveness of these interventions in reducing stress and distress in the workplace have identified barriers that hinder their applicability, such as a high dropout rate and low uptake [21,65].

The practical implications of the findings of this study are particularly relevant to improving and refining digital interventions for managing work-related stress. The integration of the TAM and the HBM helps us to understand the importance of raising awareness of the risks associated with stress prior to the development of these interventions, which can increase risk perception and consequently the perception of usefulness and the intention to use these applications. This means that not only is an effective design of mobile applications necessary, but so is placing the focus on organizations that can provide education and training to their employees about the benefits and effectiveness of mobile stress-management applications. Furthermore, to ensure that employees use these apps regularly, obtaining positive results for their well-being, organizations need to explore innovative strategies for promoting greater adherence (e.g., incentives, reminders). High app adherence not only enhances employees’ well-being but also ensures that the investment in the intervention process is effectively utilized. In addition, this study highlights the importance of perceived ease of use and emphasizes the need to invest in user experience, which is critical to reaching workers with different profiles, considering other variables that may influence both actual use and the perceived ability to do so, such as digital skill level or age [65]. Therefore, this study provides insights for the effective development of mobile applications that aim to improve the quality of the work experience, increase productivity, and reduce stress-related workplace complaints. Companies can integrate these applications as part of broader corporate wellness initiatives (e.g., programs). This not only benefits individual employees but also fosters a corporate culture that values work–life balance and occupational health.

## 6. Limitations and Future Directions

Although this study yielded promising results, some limitations should be considered when interpreting the results. First, the sample was recruited from a single region of Italy, which may limit the generalizability of the results, although they are consistent with previous studies in other regions and countries. Future studies with broader and more heterogeneous samples should replicate the results. Second, the cross-sectional design of this study provided a snapshot of the situation, but it does not allow causal inferences. Future studies could use the model proposed in this study in a longitudinal design to reduce potential bias and increase the robustness of the results. A longitudinal study could also facilitate the evaluation of the true effectiveness of mobile-based interventions to promote the management of work-related stress and worker well-being. This type of study would allow data to be collected over a longer period of time, providing insight into the long-term effects of the intervention. Finally, the variables evaluated in this study were self-reported and could bias the results. Including objective measures (e.g., actual use of the app) and considering other variables that may influence outcomes (e.g., self-efficacy) would be interesting to obtain more valid and reliable information.

## 7. Conclusions

This study integrates two important models (the TAM and the HBM) to examine how to increase the user adoption of mobile-based interventions to promote workplace health, such as stress management. The findings highlight the significant role that beliefs about health risks, PU, and PEOU play in intentions to use mobile apps to manage work-related stress. By integrating these theoretical models, researchers can gain insights into workers’ acceptance of technology and their intentions to engage in preventive health behaviors.

In summary, the findings of this study highlight the importance of a broader paradigm for the appropriate design and implementation of health-related apps in the workplace to encourage their use without sacrificing adherence. In addition, the results suggest that a focus on health risk perceptions may improve the user adoption of these digital interventions. Further experimental studies are needed to examine the efficacy of this model in mobile-based interventions to prevent and manage work-related stress.

## Figures and Tables

**Table 1:** Descriptive statistics, correlations, and Cronbach’s alpha of the considered variables.

	*r*							
	M	SD	1	2	3	4	5	6
PU	2.96	0.77	0.89					
2. PEOU	3.67	0.73	0.46 ***	0.94				
3. INT	3.05	0.85	0.72 ***	0.44 ***	0.93			
4. PSU	2.60	0.99	0.17 **	0.15 **	0.28 ***	0.91		
5. PSE	3.02	1.03	0.16 **	0.16 **	0.28 ***	0.62 ***	0.89	
6. GEN			0.14 *	0.09	0.09	0.10	0.06	
7. PGH			−0.07	−0.07	0.02	0.23 ***	0.18 **	0.07

Note. Cronbach’s alpha on the diagonal. *** *p* < 0.001; ** *p* < 0.01; * *p* < 0.05; M = mean; SD = standard deviation; PU = perceived usefulness; PEOU = perceived ease of use; INT = intention to use; PSU = perceived susceptibility; PSE = perceived severity; GEN = gender (1 = female); PGH = perceived general health.

**Table 2:** Fit indices for the model selected and the alternative models.

Model	*χ* ^2^	*df*	*p*	RMSEA	SRMR	CFI	TLI	AIC	Comp.	Δ χ^2^
One-factor model ^a^	3872.22	170	0.000	0.23	0.23	0.37	0.30	20,762.74		
Five-factor model ^b^	345.12	160	0.000	0.05	0.05	0.97	0.96	17,254.64	M1–M2	3527.10 ***
**Second-order model ^c^**	**101.52**	**48**	**0.000**	**0.05**	**0.03**	**0.98**	**0.97**	**10,193.67**	**M2–M3**	243.59 ***

Note. *df* = degree of freedom; RMSEA = root mean square error of approximation; SRMR = standardized root means square residual; CFI = comparative fit index; TLI = Tucker–Lewis index. In bold = the selected model. ^a^ All indicators load on a single factor. ^b^ All indicators load on specific factor. ^c^ PSU and PSE load on a second-order factor. *** *p* < 0.001.

**Table 3:** Standardized indirect effects for mediation models.

Standardized Indirect Effects	Estimates	SE	95% CI
PEOU → PU → INT	0.30 ***	0.05	[0.23; 0.43]
PHR → PU → INT	0.14 *	0.05	[0.04; 0.22]

Note. * *p* < 0.05; *** *p* < 0.001; SE = standard errors; 95% CI = bootstrapping lower and upper limit bias-corrected 95% confidence intervals; PEOU = perceived ease of use; PU = perceived usefulness; INT = intention to use.

## Data Availability

All data used for this study are available from the corresponding author upon reasonable request.
